# Meeting Kids Where They Are At–A Substance Use and Sexual Risk Prevention Program via Telemedicine for African American Girls: Usability and Acceptability Study

**DOI:** 10.2196/16725

**Published:** 2020-08-11

**Authors:** Cristina Lopez, Amanda K Gilmore, Angela Moreland, Carla Kmett Danielson, Ron Acierno

**Affiliations:** 1 Medical University of South Carolina Charleston, SC United States; 2 Georgia State University Atlanta, GA United States; 3 University of Texas Houston, TX United States

**Keywords:** adolescents, substance use, sexual risk reduction, telehealth, prevention programs, mobile phone

## Abstract

**Background:**

Rural African American youth lack access to drug and sexual risk–taking *prevention* programs available in more urban areas. Recent data indicate that rural youth now use substances at higher rates and at younger ages than their urban peers.

**Objective:**

This study aims to evaluate the initial usability and acceptability of a low-cost, technology-based approach to delivering effective, culturally tailored, integrated substance use disorder (SUD) and HIV risk behavior prevention programs to African American female youth to inform the use of this intervention via telemedicine for rural youth.

**Methods:**

Effective SUD prevention strategies and emotion regulation skills were integrated into an existing evidence-based HIV risk reduction program culturally tailored for African American female adolescents—Sisters Informing, Healing, Living, and Empowering (SIHLE)—and delivered to 39 African American female youth via group telehealth. The evaluation of the resulting program, 12-session SIHLEplus, was completed by 27 girls who also completed self-report measures that assessed sexual risk behaviors (eg, number of partners and age of sex initiation), substance use, exposure to traumatic events, and emotion regulation.

**Results:**

The descriptive and qualitative results of the pilot study demonstrate the initial usability and acceptability of delivering evidence-based prevention successfully via telehealth to help address health disparities in this vulnerable population.

**Conclusions:**

Although more research is needed, the findings from this study suggest that SIHLEplus has demonstrated initial usability and acceptability.

## Introduction

Substance use is common among adolescents, with 18.2%-55.7% of 8th to 12th graders using alcohol, 23.9% using cannabis, and 9% using other illicit drugs annually in 2018 [1]. Rural youth are particularly at risk for substance use [2,3], with youth in rural areas using substances such as alcohol, tobacco, and illicit drugs at higher rates [4,5] and at younger ages [5] than their urban peers. This is particularly problematic because early initiation of substance use is strongly linked with substance use disorders (SUD) in both adolescence and adulthood [6-9]. The risk for negative sequelae related to early substance use initiation in female adolescents (eg, education and employment failure, risky sexual behavior, unintended pregnancy, criminal justice system involvement, trauma exposure, mental and physical health problems) continues into adulthood [10-12], highlighting the public health impact of adolescent substance use. This is particularly a concern in low-resource areas where recovery programs are extremely limited and difficult to access. Indeed, only 1.2% of the nation’s substance abuse treatment facilities are located in small nonurban, nonadjacent counties [13]. These health care disparities in substance use services exacerbate the problems of adolescent substance use, as states with the greatest unmet need for alcohol treatment among youth aged 12-17 years are largely rural [14].

Substance use can impair sexual decision making [15], leading to a range of consequences from sexual risk behaviors, including unwanted pregnancy and sexually transmitted infections (STIs), as well as trauma exposure such as sexual assault [16]. The consequences of sexual risk behaviors disproportionately impact adolescent girls, with youth aged 15 to 24 years representing half of new STIs [17], and girls are particularly vulnerable to the consequences of STIs [18]. HIV infection is particularly problematic for African American youth living in the rural South, with an infection rate over 800% that of Whites in the same region [19]. Therefore, programs are needed to provide integrated substance use and sexual risk–taking prevention to rural African American adolescent girls.

### Adolescent Substance Use and Sexual Risk Taking

Evidence-based HIV risk reduction programs exist, which are culturally tailored for African American female adolescents. Specifically, Sisters Informing, Healing, Living, and Empowering (SIHLE) [20] is a group-based prevention program that uses ethnic and gender pride to empower African American female adolescents and provides content on HIV knowledge, communication, condom use skills, and healthy relationships. African American women who participated in SIHLE, compared with those who participated in a control group on exercise and nutrition, reported more consistent condom use, less new sexual partners, and better condom negotiation skills 1 year after the prevention program [20]. However, SIHLE does not address substance misuse despite substance use in itself being a risk factor for sexual risk behaviors. Evidence-based models of substance use prevention, including motivational enhancement therapy [21], are effective at reducing substance use among adolescents, and targeting two related health behavior constructs in an integrated manner is more effective than targeting each separately [22]. Due to the interrelated nature of substance use and sexual risk behaviors, integrated interventions are needed for African American girls who live in rural areas. Integrated interventions targeting multiple health behaviors can also address crosscutting underlying contributors to both behaviors, such as emotion regulation. Adolescent health behavior change is influenced by emotion regulatory processes on behavior and behavior change [23]. Therefore, training in problem-solving and condom use skills to modify adolescent change through skill acquisition, without any modification of the underlying system of self-regulating emotion, is shortsighted. To our knowledge, no studies have examined whether SIHLE or motivational enhancement therapy alone changes emotion regulation. However, neither intervention directly targets emotion dysregulation or provides emotion regulation skills. This is an important limitation given that both sexual risk behavior and drug use are associated with difficulties in emotion regulation among adolescents [24-26], and directly teaching emotion regulation skills is associated with changes in sexual risk behavior among adolescents [27-29]. Thus, targeting emotion regulation in an integrated intervention would not only address a factor associated with substance use and sexual risk behaviors but it may also facilitate behavior change among adolescents.

### Need to Develop Telemedicine Approaches to Reach Rural African American Girls

Fortunately, and in contrast to the lack of accessible preventive programs for substance use in rural African American adolescent girls, community-based interventions for sexual risk behavior designed for rural settings exist and show initial efficacy [30,31]. It may be particularly useful, therefore, to leverage the existing, tested infrastructure of such HIV prevention programs and incorporate substance risk reduction strategies alongside those for risky sexual behavior. Leveraging technology to deliver this service via telemedicine technology directly into participants’ communities and homes helps overcome traditional barriers to care (eg, stigma, lack of anonymity, and lack of transportation) and lack of resources (eg, no access, not enough providers to meet local needs, and no treatment space) that typically deter rural youth from successful program completion. Telemedicine has been found to be noninferior to in-person delivery of complex treatments [32] and may be an effective means to address health disparities by reducing the significant barriers associated with accessing treatment [33].

### This Study

This study examined the usability and acceptability of a telemedicine prevention program for African American rural youth that targets substance use and sexual risk behaviors (SIHLEplus). SIHLEplus integrates SIHLE [20] with motivational enhancement therapy [21] to target both substance use and sexual risk behaviors in an integrated manner using culturally tailored programming for African American adolescent girls and led by a community-accepted professional (eg, stakeholder at a community agency, near peer with established trust). In addition, emotion regulation exercises were integrated to increase the likelihood of implementing sexual risk reduction skills and substance use prevention strategies. It was hypothesized that SIHLEplus would be usable and acceptable among African American girls using a telemedicine delivery modality.

## Methods

### Overview

Quantitative and qualitative data were collected from adolescents (aged 13-18 years) recruited from local schools and community events to participate in a preventive group-telehealth intervention, SIHLEplus, focused on prevention of HIV, substance use, and other risky behaviors. Before engagement in SIHLEplus, self-report questionnaires were completed by adolescents assessing substance use, sexual risk, internalizing symptoms, and difficulties with emotion regulation. Postintervention usability interviews were also conducted with a subsample of the adolescents (n=9) who completed at least 80% (ie, 9 sessions or more) of the 12-session intervention (11 group sessions and 1 parent session) to obtain additional information about engagement factors related to content and format (eg, telehealth).

### Participants and Procedures

**Figure 1:**
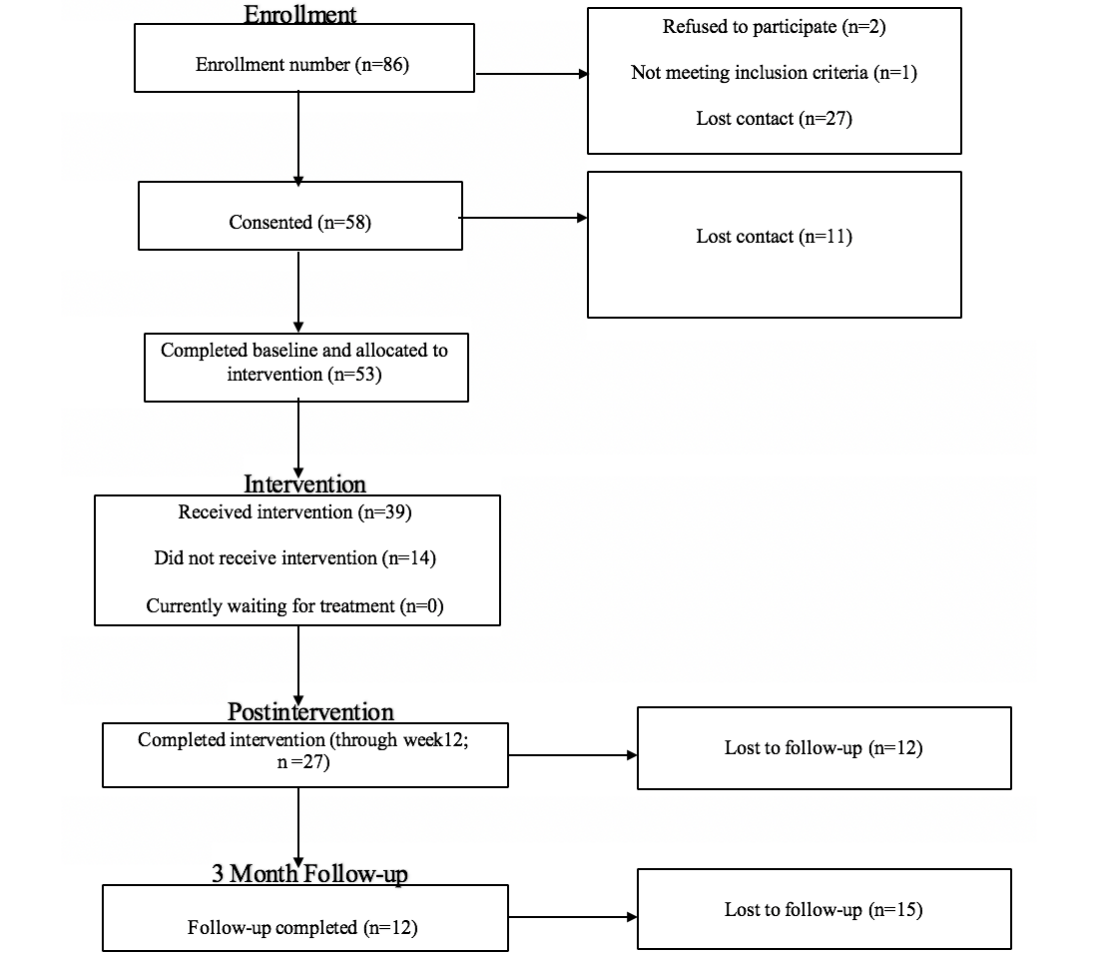
CONSORT diagram.

Participants were recruited through several community events and health fairs, referrals from a multicounty in-school case management program (14/36, 38.9% reported referrals from a school-related professional or paraprofessional), snowball recruitment (18/36, 50% of the sample reported hearing about the study from a friend or family member), and referrals from guidance counselors at several middle and high schools. Given the setting in a tricounty area of a southeastern state (South Carolina), several community events took place in rural counties and more urban areas that attracted both urban and rural populations (eg, Black Expo). Eligibility for participation included identifying as Black or African American and female, aged 13-18 years, and endorsing engagement in a health risk behavior (ie, either sexual activity in the past 12 months or reporting mild substance use activity in the previous 3 months). Reliable access to the internet was also assessed (all potential participants met internet access criteria). These pre-eligibility screening questions were completed before the consent process. After informed consent was obtained from participants and their caregivers, adolescents completed the baseline self-report questionnaires, including the drug abuse screen test (DAST)-10 instrument for substance use assessment. Consistent with other substance use prevention protocols, exclusion criteria included moderate to severe substance use problems as assessed by the DAST-10 [34]. Participants who met this exclusion criterion (n=1) were connected with an appropriate treatment agency to receive an established treatment intervention. Participants who completed the self-report questionnaires at baseline and postintervention (n=27) were adolescents who identified as Black (25/27, 92.6%) and Hispanic (2/27, 7.4%). The participants were 7th graders (2/27, 7.4%), 8th graders (6/27, 22.2%), 9th graders (5/27, 18.5%), 10th graders (4/27, 14.8%), 11th graders (4/27, 14.8%), 12th graders (2/27, 7.4%), and community college students (4/27, 14.8%). All participants identified as female, and the US census designation demonstrated that 27.3% of the sample was from a rural area, 30.3% from an urbanized area, and 42.4% from an urban cluster. Nine postintervention usability interviews (22% rural) were conducted with adolescents who had completed the web-based program. Six intervention groups were conducted over the time of the study, with groups ranging from 7 to 9 members (with the exception of the first group, which contained 2 participants). Please refer to the Consolidated Standards of Reporting Trials diagram for more details on screening and recruitment (Figure 1).

### The Intervention: SIHLEplus

SIHLEplus is an integrated intervention built from existing evidence-based HIV risk reduction program culturally tailored for African American female adolescents: SIHLE [20]. Using the empirically supported ADAPT-ITT (Assessment, Decision, Administration, Production, Topical experts, Integration, Training, Testing) model [35,36] for systematically adapting HIV behavioral programs, evidence-based substance use prevention strategies and emotion regulation skills were integrated into a telehealth-friendly SIHLEplus program. SIHLEplus complements skill acquisition of HIV prevention models with enhancement of emotion regulation skills and coping strategies to increase adolescent behavior change, resulting in the adoption of healthy HIV prevention behaviors (eg, reduced substance use). SIHLEplus consists of 11 weekly 1-hour group sessions with youth participants and 1 20-min individual session with each parent of SIHLEplus participants at some point between weeks 5 and 9 (totaling 12 weeks). All these sessions are delivered via telehealth, with participants logging in to the group virtual room by clicking on a hyperlink sent out by the group facilitator to the participant’s email address or via a text weblink. A brief session with parents was included and highlights the importance of parental communication about sex and substance use and parental monitoring, 2 central components in youth externalizing behavior treatment [37,38]. Various education delivery strategies are employed in each session, including lectures, discussions, role-play and cognitive rehearsal, skill acquisition demonstrations, and interactive games (Table 1).

**Table 1:** Table of contents for the SIHLEplus manual.

Modules, activities	
***Session One^a^:* Gender, Pride, and Self-Image**	
	*1.1 Program Intro***—**Introduce ground rules, SIHLEplus motto, and purpose of the group
	*1.2 Definition of Self-Image and Behavior***—**Discuss positive characteristics of young Black women
	*1.3 A Room Full of Sisters***—**Review poem discussing the uniqueness of Black women
***Session Two:* Influence, Relationships, and Role Models**	
	*2.1 Call Me Black Woman***—**Review poem to revisit pride in being a young Black woman
	*2.2 Magic Triangle***—**Introduce the relationship between one’s thoughts, emotions, and one’s reactions
	*2.3 Media Masquerade***—**Participants are shown a video and pictures to start the discussion of how the media can influence one’s behaviors and decisions. Emphasis is placed on how the media normalizes sex and drug use
***Session Three:* Decision Making and Peer Influences**	
	*3.1 Everyday Decision and Difficult Decision***—**Participants and group leader discuss the differences between a common decision (ie, what one should wear today) versus a more difficult decision (ie, should I have unprotected sex) and how those decisions can impact one’s life on various levels
	*3.2 What influences our Decision Making***—**Participants discuss potential influences of their decision making (ie, parents, peers, and media) and how potential influencers play a part in both one’s common (everyday) and difficult decisions
	*3.3 Identifying Situations that Involve Decisions***—**Participants discuss the benefits of one’s reactions and behaviors aligning with one’s self-image and the various options one has in how they react to an experience or situation
***Session Four:* Personal Values and Challenging Negative Views**	
	*4.1 Values: What Matters Most***—**Discuss individual values and how those values contribute to one’s decision making
	*4.2 Challenging Different Perspectives and Views***—**Discuss core beliefs, influences of core beliefs, and how core beliefs can influence our interpretation of and perspective in an experience
	*4.3 SIHLE Sistas are Special***—**Participants are reminded of why they are so special and unique. There is discussion about how SIHLE sistas are special because they know what is important to them, but most of all, they know how to care for themselves
***Session Five:* STD^b^ Knowledge**	
	*5.1 STI*^c^*Knowledge***—**Participants are given facts about STIs that are common among teens. There is further discussion about the myths and facts surrounding the behavioral causes and contributors to STIs
	*5.2 HIV/AIDS Information and Facts***—**Participants discuss knowledge about contracting HIV/AIDS, the importance of getting tested, and where testing centers are located
	*5.3 Are you at Risk***—**Participants discuss levels of risk (ie, unsafe, safe, and safer) and the behaviors that fall in line with each individual level of risk
***Session Six:* Relationships**	
	*6.1 Healthy and Unhealthy Relationships***—**Participants discuss the differences between healthy and unhealthy relationships
	*6.2 A Chain Analysis: Freeze***—**Participants discuss the importance of slowing down the sequence of stressful events in efforts to make a better decision in response to a potentially stressful situation
***Session Seven:* Self-Care and Pregnancy Prevention**	
	*7.1 Taking Care of You***—**Participants revisit the importance of self-care, more specifically self-care in reference to safer sexual behaviors
	*7.2 LIPSTICK*^d^**—**Participants learn the proper steps to appropriate condom use
	*7.3 Phenomenal Women***—**Participants review Phenomenal Women and discuss one’s sense of pride and Black beauty
	*7.4 What’s In It For You?***—**Participants reexamine the outcomes and benefits of choosing to engage in “safe” and “safer” behaviors
***Session Eight:* What is drug abuse?**	
	*8.1 What is drug abuse***—**Drug abuse is defined, and common drugs used among teens are discussed
	*8.2 Identifying the Causes of Drug Use***—**Common reasons for drug use among teens are discussed
	*8.3 Effects of Drug Abuse***—**Common effects of drug abuse and how the outcomes of drug abuse are initially associated with one’s original decision to use drugs are discussed
	*8.4 Reasons not to Abuse Drugs***—**Participants are reminded of why some teens use drugs and the benefits of not using drugs
	*8.5 Jeopardy***—**Participants are quizzed on the information they have learned thus far
***Session Nine:* Refusal Skills**	
	*9.1 KISS: Know, Indicate, State, and Stand***—**Participants discuss the importance of refusing unsafe sex and other high-risk behaviors
	*9.2 Name Game***—**Participants are taken through a simulated web-based party to discuss the outcomes of engaging in high-risk behaviors at the party
	*9.3 What Have we Learned?***—**Participants recap the information learned about the transmission of STDs and HIV
***Session Ten:* Anxiety and Anger’s Contribution to Risky Decisions**	
	*10.1 Anxiety***—**Anxiety is defined, and participants discuss how anxiety can impact behaviors and decisions
	*10.2 Explanation of Anger***—**Anger is defined, and participants discuss how anger can impact behaviors and decisions
***Session Eleven:* Communication**	
	*11.1 Three Ways to Say it***—**Participants define assertive, aggressive, and passive communication through role-playing. Participants also talk about the importance of assertive communication in refusing unsafe sexual behaviors
	*11.2 Still I Rise—*Participants review poem and discuss one’s ability to overcome obstacles
	*11.3 Graduation!* Participants are welcomed into SIHLEplus sisterhood
*Parent Module*	
	*Parent Module* provides support to parents about how to handle the topic of sex and the importance of parent-child communication and parental monitoring

^a^Text in italics is the name of the chapter used in SIHLE.

^b^STD: sexually transmitted disease.

^c^STI: sexually transmitted infection.

^d^LIPSTICK acronym stands for steps for appropriate condom use–the steps are as follows: L–Look at the expiration date; I–Inspect the condom tip to make sure it is latex or polyurethane; P–Pinch the tip of the condom to leave room for ejaculation; S–Slowly roll the condom to the base of the penis; T–Time for some action!; I–Immediately have your partner remove his penis out and away from you after he ejaculates; C–Carefully take off condom and throw it in the trash; K–Keep in mind all of these steps each and every time you have sex!

Modules of SIHLEplus cover the following topics: gender, pride, and self-image (session 1); influence, relationships, and role models (session 2); decision making and peer influences (session 3); personal values and challenging negative views (session 4); sexually transmitted disease (STD) knowledge (session 5); stress and relationships (session 6); self-care and pregnancy prevention (session 7); substance use knowledge (session 8); refusal skills (session 9); influence of anxiety and anger on behavior (session 10); effective communication (session 11); and parent module on parent-child communication and parental monitoring (session 12). Of note, the terminology used to describe a romantic partner utilizes gender-neutral language, and discussion of safe sex practices within same-sex couples is reviewed in the content. All SIHLEplus facilitators (n=3) were formally trained in the delivery of in-person SIHLE. Group facilitator training for this study focused on adaptations in SIHLEplus and coaching on the software platform.

### Measures

#### Usability and Acceptability

Initial usability and acceptability were assessed using recommendations to peers, content engagement, and delivery mode. Specifically, 3 single items were used to assess usability: “I would recommend this program to my friends,” “The SIHLEplus content was appropriate and engaging,” and “The videoconferencing format that was on my laptop or phone was easy to use.” Response choices ranged from strongly agree (1) to strongly disagree (5). Furthermore, CollaboRATE [39], a 3-item assessment of shared decision making in treatment, was used to assess the effectiveness of the SIHLEplus group facilitator. These items included the following: “How much effort was made to help you understand your health issues,” “How much effort was made to listen to what matters most to you about your health issues,” and “How much effort was made to include what matters most to you in choosing what to do next.” Response choices ranged from no effort at all (0) to every effort was made (9).

#### Baseline Risk Behaviors

Drug, alcohol, trauma, emotional regulation, and sexual risk behaviors were assessed using the DAST-10 [34,40] and the alcohol use disorders identification test (AUDIT) [41,42]; items related to sexual risk and exposure to traumatic events were assessed using the University of California at Los Angeles Posttraumatic Stress Disorder Child/Adolescent Reaction Index (UCLA-RI); and difficulties with emotion regulation were assessed using the emotion regulation questionnaire (ERQ) and difficulty in emotion regulation scale (DERS) at baseline. The DAST-10 [34] is a 10-item self-report instrument that yields an index score measuring the degree of consequences related to drug abuse. Similarly, the AUDIT is a 10-item self-report screening tool to assess alcohol consumption, drinking behaviors, and alcohol-related problems. To assess for sexual risk, questions asked are as follows: “Have you ever willingly had vaginal sex” (yes, no, prefer not to answer) and “How old were you the first time you willingly had vaginal sex?” (prefer not to answer, answer options ranged from 9 to 18 years old). The UCLA-RI [43,44] is a self-report questionnaire to screen for exposure to traumatic events in school-aged children and adolescents. The 15-item screener includes items that assess for child sexual or physical abuse, witnessing domestic or community violence, natural disasters, unexpected deaths, scary medical procedures, war, and serious accidents and an option to write in a traumatic event in an open text field. The ERQ [45] is a 10-item scale designed to measure participants’ tendency to regulate their emotions through cognitive reappraisal (CR) and expressive suppression (ES; eg, emotional avoidance). Each item has response options ranging from 1 (strongly disagree) to 7 (strongly agree). Both total scores and subscales were calculated by summing the respective questions. In addition, the DERS [46] is a 36-item self-report questionnaire that was administered to assess multiple aspects of emotion dysregulation. Response options for each of the DERS items range from 1 (almost never) to 5 (almost always). Both total scores and subscales were calculated by summing the respective questions.

### Procedure

The research protocol was approved by the institutional review board (IRB) at the affiliated university, including a written informed consent process of all participants. The use of HIPAA-compliant Adobe Connect for the group telehealth format was used because the functionality allowed for multiple user logins from various types of devices (eg, laptops, tablets, and smartphones). The institution (Medical University of South Carolina) had already used the HIPAA-compliant Adobe Connect platform for telehealth services in a clinical manner, but IRB approval was sought for research use of Adobe Connect [33]. Before completion of the self-report questionnaires, informed consent and parental consent (aged under 18 years) were obtained. Participants (ie, adolescents) were compensated with Walmart gift cards following completion of web-based (Research Electronic Data Capture) self-report questionnaires at baseline (US $20) and postintervention (US $30).

### Statistical Analyses

#### Quantitative Analyses

Quantitative analyses were conducted using SPSS 25. Descriptive statistics were completed to assess satisfaction with SIHLEplus (Table 2) and to describe the baseline characteristics of the sample.

**Table 2:** Descriptive statistics of usability findings.

Usability items	Indicated agree or strongly agree, n (%)	Value, mean (SD)
I would recommend this program to my friends	9 (100)	1.22 (0.55)
The SIHLE^a^plus content was appropriate and engaging	9 (100)	1.22 (0.43)
The videoconferencing format on my laptop or phone was easy to use	7 (77.8)	2.06 (1.00)
Participant ratings of group facilitator	N/A^b^	8.39 (0.73)

^a^SHILE: Sisters Informing, Healing, Living, and Empowering.

^b^N/A: not applicable.

#### Qualitative Analyses

Usability interviews were conducted with participants post intervention (ie, who had completed the full intervention) utilizing semistructured questions, with prompts based on the participant’s responses. Content addressed usability and acceptability of the intervention and software platform (a list of semistructured questions is shown in Multimedia Appendix 1). Interviews lasted approximately 45 min and were transcribed. NVivo 12.0 software was used for data management and analysis. Analyses of the focus group data consisted of qualitative content analysis [47] informed by grounded theory [48]. Grounded theory explores participants’ unique perspectives via the identification of themes or patterns that naturally emerge from the data and the systematic classification of these themes [49]. Specifically, a 3-step inductive approach was utilized, in which the interview responses (ie, raw data) were carefully examined to develop a comprehensive codebook to capture all possible themes emerging from the data [50]. The codebook was then used by 2 independent coders to code and analyze each participant’s responses to the interview questions [47,49]. Coders were able to apply more than one code to participant responses if applicable. Interrater discrepancies were discussed and resolved by 2 independent coders. Finally, themes were refined, merged, or subdivided into subthemes. Percentages reported in the findings reflect the percentage of times that the specific themes were mentioned by all participants in the interviews.

## Results

### Descriptive Statistics

At baseline, 12 (25.5%) participants indicated that they had engaged in consensual vaginal sex during their lifetime. Responses for the age at the first consensual sexual intercourse ranged from 12 to 18 years. The mean age for engaging in consensual sex was 15 (SD 1.81) years. Participant responses indicated that 41.7% of those who had consensual vaginal sex reported the age of initiation of willing vaginal sex at 14 years or below (n=5). Although average scores on the DAST-10 (mean 0.33, SD 0.72) and AUDIT (mean 0.94, SD 1.43) indicated low drug and alcohol use, a total of 9 (19%) participants indicated that they had used drugs. Scores endorsed on the DAST-10 showed that 10.4% of the sample scored 2 or above, indicating problems consistent with a mild SUD. In response to exposure to traumatic events, the sample reported a range of 0 to 11 (out of potential 15), with a mean of 3.14 (SD 2.31) traumatic events experienced in their lifetime. Frequencies demonstrated that more than 50% of the sample reported exposure to 3 or more traumatic events in their lifetime, with 16.7% endorsing 6 or more traumatic events. The ERQ yielded a mean of 27.15 (SD 8.74) for the CR subscale, and the ES subscale yielded a mean of 15.29 (SD 6.00), scores higher than the average CR and ES scores obtained from a community sample of girls aged 10 to 18 years (mean 21.47, SD 3.81 and mean 10.18, SD 2.97, respectively [45]). Emotion regulation as measured by the DERS yielded a total mean score of 95.35 (SD 24.11), a score that is higher than the average of 80.2 (SD 23.4) in a community sample of female adolescents (higher scores indicated more difficulty with emotion regulation [51]).

### Initial Usability and Acceptability Findings

Overall, participants rated the usability of SIHLEplus positively. The majority of participants indicated that they would recommend SIHLEplus to their friends, that the content was appropriate and engaging, and that the videoconferencing format was easy to use (Table 2). Furthermore, participants were able to engage with the group facilitator over the videoconferencing format and reported high facilitator engagement and effort (Table 2).

### Qualitative Results

Four overarching themes, each with their own subthemes, emerged from the participants’ responses during the interviews. Each is described below with representative quotes provided throughout for illustrative purposes.

#### Program Usage

Discussion of program usage occurred on 15 occasions throughout the 9 interviews (14.7% of all content). Program usage included discussion of place of log-in (46.7% within this theme), including at home, in their bedroom, or in the car; ability to log in to the site (40% within this theme), including difficulties with log-in on first usage, ongoing difficulty with log-in, log-in taking a long time, or always being able to log-in; and technical concerns (13.3% within this theme), including some technical issues that needed repair. Specific comments that participants made about program usage included:

I logged in at home, in my room where I usually do my homework.

I did have problems a couple of times because of my phone. Sometimes you can’t copy and paste the link into the bar. Sometimes I had to type out the whole link.

#### Strengths of the Program

Conversation regarding strengths of the program occurred on 54 occasions throughout the interviews (52.9% of all content). The reported strengths of the program included the relationships that were formed with other participants (11.1%), such as feeling comfortable with group members, liking that they were able to build relationships with similar-aged females, and liking the intimacy of the group. In addition, comments about new information learned in the group arose in 31.5% of interviews within this theme, including communication skills, safe sexual practices (condom use), positive relationship skills, STDs, and decision making. Adolescents commented on the use of skills learned in the program (53.7% within this theme), such as communication skills, safe sexual practices (condom use), positive relationship skills, problem-solving skills, meditation, coping skills or how to handle stress, decision making, and keeping sex education conversations ongoing with adolescents. Finally, adolescents liked the web-based platform (3.7% within this theme), reporting that it was more convenient and that they felt more open sharing private information on the web than they would have been in an in-person setting. Specific comments about the strengths of the program included:

Liked other girls my age because we all had the same ideas. Well, we were different, but we were all in the same boat and could relate easier.

It was pretty comfortable. I mean other than the fact that I knew one person in the group but I wasn’t, like, scared to talk about anything.

When it came down to the sexual stuff, when done with the STD talk, it had me sit and think, we were always having sex without a condom. I know he was locked up, but we need to practice safe sex, for STD but also to prevent pregnancies.

Used some skills to communicate better and some skills with best friend.

I also use the meditating when I am too frustrated. I don’t know why it helps, when I felt my temperature rising.

#### Limitations of the Program

Discussions of program limitations occurred on 5 occasions throughout the 9 interviews (4.9% of all content). Overall, participants noted that they would prefer if the program was offered in person (60% within this theme), that they had to miss some sessions because of other commitments (20% within this theme), and that the group setting made it difficult to share personal information (20%). Some specific comments about the limitations of the program included:

It was just a different way of learning, but I prefer to sit down in a room. I think I like that better.

I said less things in a group. I’m not up and go get it with other people...depends on how many people I knew before giving out personal information.

I didn’t like that I missed sometimes due to conflicts in my schedule.

#### Sharing Information About the Program

Conversation about sharing information about the program occurred on 28 occasions throughout the 9 interviews (27.5% of all content). Specifically, participants reported that they would recommend the program to a friend (14.3% within this theme) or talk with their parents about the program (17.9% within this theme). Regarding sharing information with others (60.7% within this theme), 24% stated that they had shared information with others, 18% reported that they would feel comfortable sharing information but had not yet, 12% stated that they had not shared because they did not want to, 18% did not share because they did not think it was necessary, and 18% reported that they would not feel comfortable sharing information with others. Specific comments about sharing information with others included:

I showed my best friend how to use a condom. We were having girl talk about sex and how her boyfriend doesn’t use condoms. She’s on birth control and I told her birth control isn’t 100%, but I showed her with paper and pinch the tip. Have it folded like a hat (not like a baby's bottle because you don’t want babies). She didn’t know you could use a condom backwards.

I mean like, the friends I talk to, don’t really engage in the things we learned about so it wasn’t necessarily helpful but it was good information to have, if that makes sense.

## Discussion

### Principal Findings

This study assessed the usability and acceptability of SIHLEplus, a telemedicine prevention program for African American youth that targets substance use and sexual risk behaviors. Findings from this initial usability and acceptability pilot using both quantitative and qualitative results from African American adolescent girls indicated that the content was engaging and easy to use and that they would recommend the program to their friends. Furthermore, recruitment numbers show initial interest and need. Although the sample was not predominately rural, the findings from this suggest that SIHLEplus can be delivered via telemedicine in an acceptable manner and has important implications for rural youth. This is an important finding given that substance use is associated with sexual risk behaviors [15] and rural African American youth are at particular risk for substance use [2,3].

The participants reported several strengths of SIHLEplus. Participants indicated that they were able to form relationships with other participants via the telemedicine platform and that they were able to learn helpful skills. Notably, the adolescents indicated that they were more willing to share private information via telemedicine than they would have in an in-person treatment modality. This was surprising because the telemedicine delivery was only intended to assist with delivery of SIHLEplus to rural youth and not intended to enhance the treatment effects or uptake. However, this is consistent with the literature suggesting that individuals are more likely to report socially unacceptable behaviors via computer-based surveys than in-person surveys [52]. It is possible that the telemedicine platform allowed them to distance themselves from social acceptability pressures that may exist within in-person group settings. Future work is needed to test the efficacy of this SIHLEplus on substance use, sexual risk, and emotion regulation outcomes among rural youth.

Participants, in general, indicated that they would share the information learned in the program with others or that they would recommend the program to their peers. Specifically, about one-fourth of the adolescents indicated that they had already shared the skills they learned in the program, and almost all of the adolescents (94%) indicated that they would recommend the program to their friends. These findings indicate the initial usability and feasibility of SIHLEplus. Furthermore, willingness to talk with peers about these topics indicates that this does not only have the potential to reduce substance use and sexual risk behavior among those who participated, but their peers as well. Given that social norms are a strong predictor of both substance use and sexual behaviors among youth [53], this unexpected finding suggests that future work should examine the social network impacts of SIHLEplus in addition to the individual changes in substance use and sexual risk behavior.

Despite the initial acceptability and usability of SIHLEplus, there were some factors that may need to be considered before conducting a large clinical trial to test its efficacy. First, some participants indicated that they had some difficulty participating in the program because of commitments with after-school activities. Given the noted positive effects of extracurricular activities and endorsing school pride in adolescent academic achievement [54,55], the study team supported participants’ requests to be removed from the intervention. It is possible that SIHLEplus would be better adopted in states where sex education was more accepted and potentially have it as part of the school curriculum or as an extra credit activity for some courses. It is also possible that the development of a mobile health app would assuage this concern, given that it would allow adolescents to access the intervention at their convenience. However, the participants did indicate that they enjoyed the relationships formed, so the group telemedicine component may be a crucial piece of the intervention. Future work is needed to determine the feasibility of SIHLEplus given the time constraints of adolescents. Specifically, future work is needed to determine the feasibility of SIHLEplus among *rural* African American girls. It is possible that there may be differential time constraints among rural youth, and this should be considered before conducting a large-scale randomized clinical trial.

The provision of prevention and treatment through telemedicine to high-risk populations may be a useful tool to use in conjunction with treatment for mental health disorders. In the sample in this study, there was particularly high endorsement of exposure to traumatic events. Given the strong association between exposure to traumatic events, substance use, and sexual risk behaviors [15,56-58], it may be worthwhile to consider if prevention programs like this are effective at reducing exposure to subsequent potentially traumatic events. If treatment for posttraumatic stress disorder is needed, it may be possible to address posttraumatic stress symptoms in conjunction with substance use and sexual risk behaviors for rural African American youth. Emotion regulation has also been identified as a risk factor for youth who have been exposed to a traumatic event, so it is not surprising that many youth would benefit from SIHLEplus for externalizing symptoms (sexual risk reduction and substance use) and internalizing symptoms (anxiety related to traumatic stress).

### Strengths and Limitations

Strengths of SIHLEplus and this usability and acceptability study include the innovation of this integrated prevention program delivered via telemedicine, allowing for participation from an at-risk group that typically does not have access to prevention programming. This study demonstrated that the internet or smartphone access as a study criterion did not lead to an ineligibility status for any interested potential participant. Although rural residence was not part of the inclusion criteria for this initial usability step, all participants identified as an ethnic or racial minority and thus are considered underserved populations accessing behavioral health services. Given the false misconception that rural and underserved families may not have access to resources for telemedicine delivery, the findings of this pilot study can be used to justify additional delivery of medical and health information via telemedicine.

This study only assessed the initial usability and acceptability of SIHLEplus; its feasibility and efficacy need to be established before implementation. Therefore, an important next step includes assessing both feasibility and intervention effects among at-risk rural African American adolescent girls. Consistent with approaches used to define the rural HIV epidemic in South Carolina [59], rural areas will be defined using a zip-code approximation version of the rural urban commuting area (RUCA) codes to recruit participants from RUCA codes 4 to 10 (ie, rural areas, *zip-code RUCA approximation* [60]).

Although this study establishes initial usability, more studies are needed to ensure that the program can be delivered to formally defined rural populations. Subsequent feasibility studies with larger samples may look into potential differences in usability themes and engagement from rural- and more urban-residing adolescents. Although rural and urbanized areas and urban clusters were helpful descriptions for the initial stages of usability, future studies could also collect socioeconomic data to ensure generalizability across several rural subpopulations. Furthermore, a large randomized clinical trial assessing the effects of SIHLEplus on sexual risk behaviors, substance use, and emotion regulation is warranted.

### Conclusions

The findings from this study provide initial usability and acceptability for an integrated substance use, sexual risk, and emotion regulation program for at-risk African American adolescent girls delivered via telemedicine. Although future work is needed to determine feasibility and efficacy, preliminary findings established the initial usability and acceptability of SIHLEplus.

## Appendix

Multimedia Appendix 1 List of semistructured questions for usability interviews.

## Abbreviations

AUDIT: alcohol use disorders identification test
CR: cognitive reappraisal
DAST: drug abuse screen test
DERS: difficulty in emotion regulation scale
ERQ: emotion regulation questionnaire
ES: expressive suppression
IRB: institutional review board
PI: principal investigator
RUCA: rural urban commuting area
SIHLE: Sisters Informing, Healing, Living, and Empowering
STD: sexually transmitted disease
STI: sexually transmitted infection
SUD: substance use disorder
UCLA-RI: University of California at Los Angeles Posttraumatic Stress Disorder Child/Adolescent Reaction Index
