# Supplementary material for: Meeting Kids Where They Are At–A Substance Use and Sexual Risk Prevention Program via Telemedicine for African American Girls: Usability and Acceptability Study
Source: J Med Internet Res. 2020 Aug 11;22(8):e16725. doi: 10.2196/16725 (PMC7448181; doi:10.2196/16725)
Supplement: Multimedia Appendix 1 [file jmir_v22i8e16725_app1.docx]

**Appendix A. List of semi-structured questions for Usability Interviews**

1. How was your experience in the study? Describe what you liked and disliked.

2. When you logged in for group, where were you normally located? Home? Library? School?

3. Did you have any trouble logging in for group? If so, please explain.

4. Would you recommend this group to a friend?

5. Did you share any information that you may have found helpful with any of your friends?

6. Did you share any information that you may have found helpful with your parents?

7. Have you use any of the skills that you learned in group?

8. Did you share any private information about yourself during the group sessions?

9. Did you feel like the group sessions were a comfortable place to share private information about yourself?

10. Did you have any trouble accessing the survey questions?

11. Did you have any trouble understanding the survey questions?

12. Were the instructions given to complete the survey easily understood?

13. In what ways can we improve the Healthy Relationships Program?

14. Was any information you learned in the group new information? If so, what kind of information?
